# A Rare Paronychia with Superinfection with *Prevotella bivia* and *Staphylococcus haemolyticus*: The Importance of Early Microbiological Diagnosis

**DOI:** 10.3390/pathogens9120999

**Published:** 2020-11-29

**Authors:** Jessica Grande-Del-Arco, María Dolores Jimenez-Cristino, Raquel García-de-la-Peña, Emilio Fernández-Espejo, Antonio Córdoba-Fernández

**Affiliations:** 1 Facultad de Enfermería, Fisioterapia y Podología, Universidad Complutense de Madrid, 28040 Madrid, Spain; jessicagrandedelarco@gmail.com; 2 Departamento de Podología, Universidad de Sevilla, 41009 Sevilla, Spain; mjimenez45@us.es (M.D.J.-C.); raquelgp@us.es (R.G.-d.-l.-P.); 3 Reial Acadèmia de Medicina de Catalunya, 08001 Barcelona, Spain; efespejo@us.es

**Keywords:** paronychia, mixed infections, anaerobic, *prevotella bivia*, bacterial resistance

## Abstract

*Prevotella bivia* is an anaerobic, gram-negative bacillus which naturally thrives in the human vagina, and is usually related to vaginal tract infections. However, this microorganism can also cause infections in other body locations. Infections with *Prevotella bivia* are frequently severe due to the risk of osteomyelitis and the lack of good protocols for adequate therapeutic management. *Staphylococcus haemolyticus* infection is one of the most frequent etiological factors of nosocomial infections, which hasthe ability to acquire multiple resistance against antimicrobial agents. We report a rare case of foot and hand paronychia with superinfection of *Prevotella bivia* and *Staphylococcus haemolyticus*. We highlight the importance of early microbiological diagnosis, and proper therapeutic management to avoid the risk of complications and the development of bacterial resistance to antibiotics.

## 1. Introduction

Paronychia is a common clinical problem that is seen in the fingers and toes and can occur acutely or chronically. Acute infection usually arises as result of bacterial growth, and it is normally associated with severe pain and hypersensitivity at the nail folds that is caused by pus buildup. Clinically, the infection occurs with nail thickening, onycholysis and discoloration accompanied by redness of the eponychium. Acute paronychia is usually the result of minor lesions that are usually associated with onychophagy, aggressive manicure and pedicure or sustained exposure to toxic chemicals. Paronychia is three times more frequent in women than men, possibly because of more nail manipulation in women [1,2,3,4]. *Staphylococcus aureus* is the most prevalent pathogen in acute paronychia and is responsible for 50–80% of cases, although *Streptococcus pyogenes, Pseudomonas pyocyanea* and *Proteus vulgaris* can cause paronychia as well [2]. Chronic paronychia is less frequently seen, and it is considered as a type of onychomycosis, where *Candida albicans* plays a leading role as pathogen, and which usually occurs in patients with diabetes, psoriasis or pemphigus [5].

Superinfections with a mixture of aerobic and anaerobic organisms are not infrequent in paronychia [6,7]. Several prevalence studies reveal an increased incidence of superinfections with rare gram-negative and gram-positive anaerobic microorganisms of enteric or genital origin, such as *Prevotella* species [8,9].

*Prevotella bivia* (PB) is an unpigmented gram-negative bacterium that is a member of the commensal flora in humans, mostly in vaginal mucosa. Its growth and pathogenicity are favored by an excess of estrogens or the synergistic cooperation of other aerobic microorganisms. PB thrives in the vaginal tract, and is associated with bacterial vaginosis, pelvic inflammation disease and perianal abscesses [10,11,12]. PB has been isolated together with aerobic organisms in mixed infections on nails, oral cavity, face and neck [6,13,14,15].Many authors report that mixed infections where PB participates can induce severe complications such as necrotizing fasciitis, osteomyelitis or septic arthritis, even more if therapeutic management is poor [7,16,17,18,19]. Experimental studies in animal models have confirmed the pathogenic potential of PB to trigger severe infection and induce tissue destruction [20].

*Staphylococcus haemolyticus* (SH) is one of the coagulase-negative staphylococcus genera that inhabits the skin as a commensal pathogen. SH is prevalent both at hospital facilities and hands of healthcare workers and it is increasingly implicated in opportunistic infections in hospitalized patients [21]. The most important factor might be the ability to acquire multi-resistance against antimicrobial agents, even glycopeptides [22].

To date, no cases of paronychia due to anaerobic pathogens with superinfection by coagulase-negative staphylococci have been reported. We herein report a case of acute paronychia with PB and posterior superinfection with SH that was multi-resistant to several antimicrobial group agents. Before this report, an informed consent form was obtained from the patient and the protocol was approved by Área Clínica de Podología de la Universidad de Sevilla. 

## 2. Case Presentation

A 38-year-old Brazilian woman came to podiatry consultation with a previous diagnosis of onychomycosis on the first toe of both feet, on the fifth finger of the left hand and on the fourth finger of the right hand, all caused by *Trichophytum rubrum*. The patient did not refer to previous episodes of trauma but reported having undergone a manicure and pedicure in her home country. At physical examination, a purulent exudates was observed under the nail bed of the affected toes accompanied by pain, erythema and elevated local temperature. Onycholysis and subungual hyperkeratosis were observed in the first toe of the right foot, and they were compatible with total dystrophic onychomycosis associated with dermatomycosis located at interdigital spaces. In accordance with the previous diagnosis of onychomycosis, topical treatment (cyclopirox, 80 mg nail varnish, one application per day) on the nail plates of both toes was established. The patient was sent to the dermatology service for a more accurate diagnosis. The first bacterial culture made from the exudate of the nail of the fifth finger of the right hand, tested negative, and the patient was diagnosed with *tinea unguium*, being treated with itraconazole (100 mg/day per os). After four weeks of treatment, the patient observed thatthe pain and exudate continued, and decided to stop taking the oral antifungal treatment.

**Figure 1:**
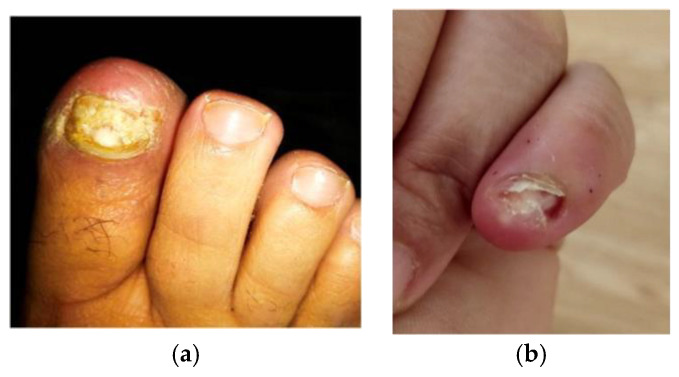
(**a**)Paronychia with total dystrophic onychomycosis on the in the first toe of the right foot; (**b**) Paronychia with onycholysis and exudate on the nail bed of the fifth finger of the left hand can be observed.

One week after leaving therapy, she again consulted the podiatrist, presenting an exudate on the nailbed of the first toe of her right foot, quite similar to what was observed in her hands (Figure 1a,b). A sample was taken for microbiological culture and antibiogram, and empirical antibiotic therapy was established with metronidazole (500 mg) plus cephalexin (500 mg), every 8 h per os. The result of the microbiological culture showedthe presence of *PB* growth; however, an antibiogram could not be obtained, after which empirical treatment with moxifloxacin was established (400 mg per day, two weeks).

**Figure 2:**
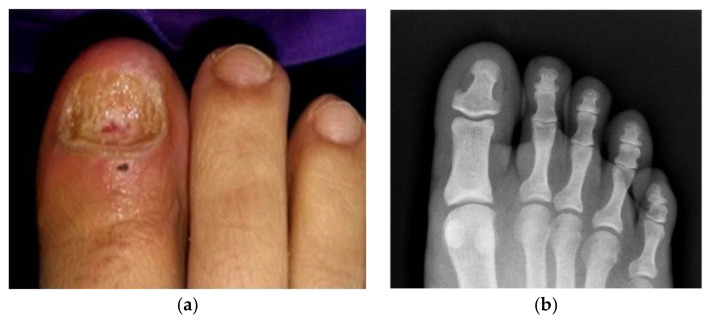
(**a**) Appearance of nail bed after debridement. The image shows the presence of exudate. (**b**) Dorsoplantar radiography did not reveal the existence of any lytic image in the distal phalanx of the first toe.

During this period, the infectious disorder evolved favorably until remission. However, at 25 days after the start of the second treatment, the patient consulted due to exacerbation of the clinical picture with a new infectious outbreak characterized by severe pain on the 5th finger of the hand, and on the first toe of the right foot. As consequence, the toenail was debrided, and the purulent content was drained. A new sample for microbiological culture was taken from the first toe. In addition, to rule out the possibility of bone involvement, a dorsoplantar radiography was performed that did not reveal the existence of any osteolytic lesion (Figure 2a,b).

**Figure 3:**
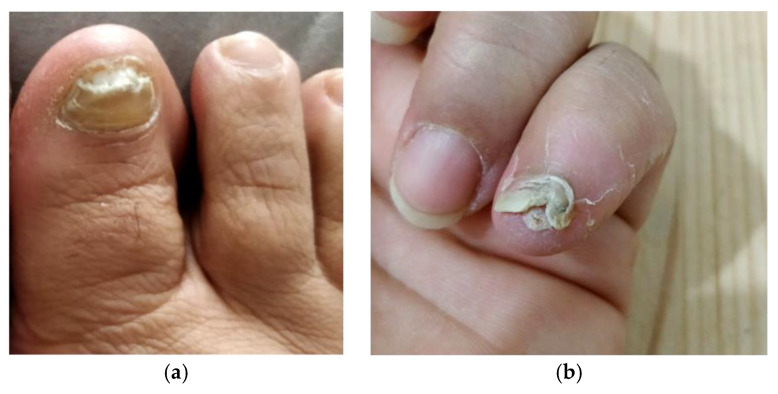
(**a**) Dismorphic appearance of the nail without signs of paronychia; (**b**) No signs of infection can be seen inthe fifth finger after two months of treatment with rifampicin.

Bacterial culture showed abundant growth of *Staphylococcus haemolyticus* (SH), and the results of the antibiogram showed resistance to penicillin, cloxacillin, quinolone and clindamycin, and a positive response to rifampicin (minimal inhibitory concentration or MIC < 0.5 mg/liter). In this second culture, no growth of PB was observed. Treatment with rifampicin (600 mg/day) was established. After two months of treatment, the purulent exudate and other clinical signs indicative of infection progressively went away (Figure 3a,b).

## 3. Discussion

Feet and hands are one of the most unique and heterogeneous microbial niches in the human body. Several factors influence the microbiome profile on fingernails and toenails such as diet, hygienic and cosmetic habits. It is also known that during hygienic procedures, in particular showers, the foot and hand microbiome is supplemented with microorganisms that normally colonize other parts of the body, including intestinal and genital bacteria [23,24,25]. These environmental circumstances, together with the disturbance of the microbiome after repeated microtraumas, facilitate the development of paronychia, where *Prevotella* species could play a significant role. As part of the normal human microbiome, hundreds of anaerobic species can be found that, under certain circumstances, can cause aggressive infections and induce bacterial resistance to antimicrobials [9,26].This is the case with PB-associated infections that may cause abscesses in different location. PB and other *Prevotella* species contain endotoxins and *lipopolysaccharides* on their cell wall which are capable to act as powerful antigens that can lead to tissue destruction. On the other hand, the capacity of PB to associate with aerobic bacteria may explain its ability to persist after the first bacterial infection remitted [6].

As in the case herein reported, when an infection by anaerobic species is suspected and a good response to treatment with conventional antimicrobials does not occur, culture and antibiogram should be rapidly performed. Thus, starting with specific treatment would permit avoiding bacterial complications and resistances. Many epidemiological studies of the efficacy of antibiotic treatment of anaerobic infections, especially *Prevotella* species, *Fusobacterium* and *Veillonella*, highlight the growing import of bacterial resistance to antimicrobials. Most anaerobic organisms are sensitive to meropenem and metronidazole, but the well-known high rate of resistance to clindamycin is of concern [27]. To date, as regards the empirical approach for anaerobic infections, metronidazole is the drug of choice. For a long time, it was considered that acquired resistance to this antibiotic was rare among anaerobic pathogens, despite its extensive use. However, recent studies have shown that this resistance is not uncommon among organisms such as PB [28].The resistance of anaerobic bacteria and multi-resistance of pathogens is currently a growing problem; however, in the present case, we consider that the treatment established for 9 days with the combination of metronidazole and cephalexin was effective in eliminating PB infection.

In a second microbiological culture, abundant growth of SH was isolated and antibiotic resistance was observed to penicillin, cloxacillin, quinolone and clindamycin. The results of the antibiogram forced treatment with rifampicin, a broad-spectrum antibiotic. SH is, after *Staphylococcus epidermidis*, the second most frequently isolated coagulase-negative staphylococcus from clinical cases, notably from blood infections, including sepsis. SH has shown to be a pathogen with important virulence because the presence of various enzymes, cytolysins and surface substances. Currently the key role in the evaluation of a threat posed by SH is its multiresistance [21,22,29]. In the case herein reported, SH acted as a pathogen with important virulence which readily acquired the ability to develop multi-resistance against many antimicrobial agents.

In the reported case, there was not a mixed infection because both pathogens did not grow together in culture, and as consequence, they did not act synergistically. However, the resistance of the multi-resistant microorganism forced us to start treatment with a broad-spectrum antibiotic. Studies have shown that the presence of various enzymes, cytolysins and surface substances affects the virulence of SH.

## 4. Conclusions

Early and accurate diagnosis is crucial for favorable remission of PB infections, particularly in hand and foot paronychia. These infections tend to progress easily to deep tissue planes with the consequent risk of bone involvement. At present, there is no consensus on protocols for the optimal treatment of PB-related periungual infections. We consider that the therapeutic approach should be based on two principles: first, the modification of the medium to slow microbial proliferation, and second, the use of appropriate antimicrobial agents. Modification of local conditions can be achieved with surgical incision of the nail, adequate drainage and decompression of edematous periungual tissues, with the aim of improving local circulation. This set of actions together with microbiological cultures and antibiograms will allow the early introduction of efficacious antimicrobial treatment. We consider that the use of broad-spectrum antibiotherapy should be discarded when paronychia caused by anaerobic organisms is suspected because this approach promotes the appearance of bacterial resistance.

## References

[1] Rigopoulus D., Larios G., Gregorious S., Alevizos A. (2008). Acute and chronic paronychia. Am. Fam. Physician.

[2] Leggit J.C. (2017). Acute and chronic paronychia. Am. Fam. Physician.

[3] Roberge R.J., Weinstein D., Thimons M.M. (1999). Perionychial infections associated with sculptured nails. Am. J. Emerg. Med..

[4] Brook I. (1993). Paronychia: A mixed infection. Microbiology and management. J. Hand Surg. Br..

[5] Tosti A., Piraccini B.M., Ghetti E., Colombo M.D. (2002). Topical steroids versus systemic antifungals in the treatment of chronic paronychia: An open, randomized double-blind and double dummy study. J. Am. Acad. Dermatol..

[6] Mirza A., Bove J.J., Litwa J., Appelbe G. (2012). Mixed Infections of the paronychium with *Prevotella bivia*. J. Hand Microsurg..

[7] Riesbeck K. (2003). Paronychia due to *Prevotella bivia* that resulted in amputation: Fast and correct bacteriological diagnosis is crucial. J. Clin. Microbiol..

[8] Weinzweig N., Gonzalez M. (2002). Surgical infections of the hand and upper extremity: A county hospital experience. Ann. Plast. Surg..

[9] Novak A., Rubic Z., Dogas V., Goic-Barisic I., Radic M., Tonkic M. (2015). Antimicrobial susceptibility of clinically isolated anaerobic bacteria in a University Hospital Centre Split, Croatia in 2013. Anaerobe.

[10] Onderdonk A.B., Delaney M.L., Fichorova R.N. (2016). The Human microbiome during bacterial vaginosis. Clin. Microbiol. Rev..

[11] Kostov S., Slavchev S., Dzhenkov D., Strashilov S., Yordanov A. (2020). An unusual case of fulminant generalized peritonitis secondary to purulent salpingitis caused by *Prevotella bivia*—Case report with literature review. Germs.

[12] Masadeh M., Hossain S., Dunkelberg J., Gerke H. (2016). Purulent proctitis caused by *Prevotella bivia* in a homosexual male. ACG Case Rep. J..

[13] Schindl A., Schön H. (1999). Foot infection with *prevotella bivia*, P. oralis and P. loescheii after wound licking. J. Med. Microbiol..

[14] Tanner A., Stillman N. (1993). Oral and dental infections with anaerobic bacteria: Clinical features, predominant pathogens, and treatment. Clin. Infect. Dis..

[15] Băncescu G., Dumitriu S., Băncescu A., Preoteasa E., Skaug N. (2004). Prevotella species involved in the onset of superficial face and neck abscesses. Bacter. Virus Paraz. Epidemiol..

[16] Salman S.A., Baharoon S.A. (2009). Septic arthritis of the knee joint secondary to *Prevotella bivia*. Saudi Med. J..

[17] Alegre-Sancho J.J., Juanola X., Narvaez F.J., Roig-Escofet D. (2000). Septic arthiritis due to *Prevotella bivia* in a patient with rheumatoid arthritis. Jt. Bone Spine.

[18] Laiho K., Kotilainen P. (2001). Septic arthritis due to *Prevotella bivia* after intra-articular hip joint injection. Jt. Bone Spine.

[19] Fihman V., Raskine L., Petitpas F., Mateo J., Kania R., Gravisse J., Resche-Rigon M., Farhat I., Berçot B., Payen D. (2008). Cervical necrotizing fasciitis: 8-years’ experience of microbiology. Eur. J. Clin. Microbiol. Infect. Dis..

[20] Mikamo H., Kawazoe K., Izumi K., Watanabe K., Ueno K., Tamaya T. (1998). Studies on the pathogenicity of anaerobes, especially *Prevotella bivia*, in a rat pyometra model. Infect. Dis. Obstet. Gynecol..

[21] Eltwisy H.O., Abdel-Fattah M., Elsisi A.M., Omar M.M., Abdelmoteleb A.A., El-Mokhtar M.A. (2020). Pathogenesis of *Staphylococcus haemolyticus* on primary human skin fibroblast cells. Virulence.

[22] Czekaj T., Ciszewski M., Szewczyk E.M. (2015). Staphylococcus haemolyticus—An emerging threat in the twilight of the antibiotics age. Microbiology.

[23] Adamczyk K., Garncarczyk A., Antończak P., Wcisło-Dziadecka D. (2020). The foot microbiome. J. Cosmet. Dermatol..

[24] Rayan G.M., Flournoy D.J. (1987). Microbiologic flora of human fingernails. J. Hand Surg. Am..

[25] Wolf E.W., Hodge W., Spielfogel W.D. (1991). Periungual bacterial flora in the human foot. J. Foot Surg..

[26] Brook I. (2016). Antimicrobials therapy of anaerobic infections. J. Chemother..

[27] Rodloff A.C., Dowzicky M.J. (2018). In vitro activity of tigecycline and comparators against a European collection of anaerobes collected as part of the Tigecycline Evaluation and Surveillance Trial (T.E.S.T.) 2010–2016. Anaerobe.

[28] Alauzet C., Mory F., Teyssier C., Hallage H., Carlier J.P., Grollier G., Lozniewski A. (2010). Metronidazole resistance in Prevotella spp. and description of a new nim gene in Prevotella baroniae. Antimicrob. Agents Chemother..

[29] Barros E.M., Ceotto H., Bastos M.C., Dos Santos K.R., Giambiagi-Demarval M. (2012). Staphylococcus haemolyticus as an important hospital pathogen and carrier of methicillin resistance genes. J. Clin. Microbiol..

